# Supplementary material for: Functional correlates of clinical phenotype and severity in recurrent SCN2A variants
Source: Commun Biol. 2022 May 30;5:515. doi: 10.1038/s42003-022-03454-1 (PMC9151917; doi:10.1038/s42003-022-03454-1)
Supplement: Supplementary file 2 — Supplementary Information [file 42003_2022_3454_MOESM2_ESM.pdf]

SUPPLEMENTARY INFORMATION

Functional correlates of clinical phenotype and severity in recurrent *SCN2A* variants

---

SUPPLEMENTARY FIGURES

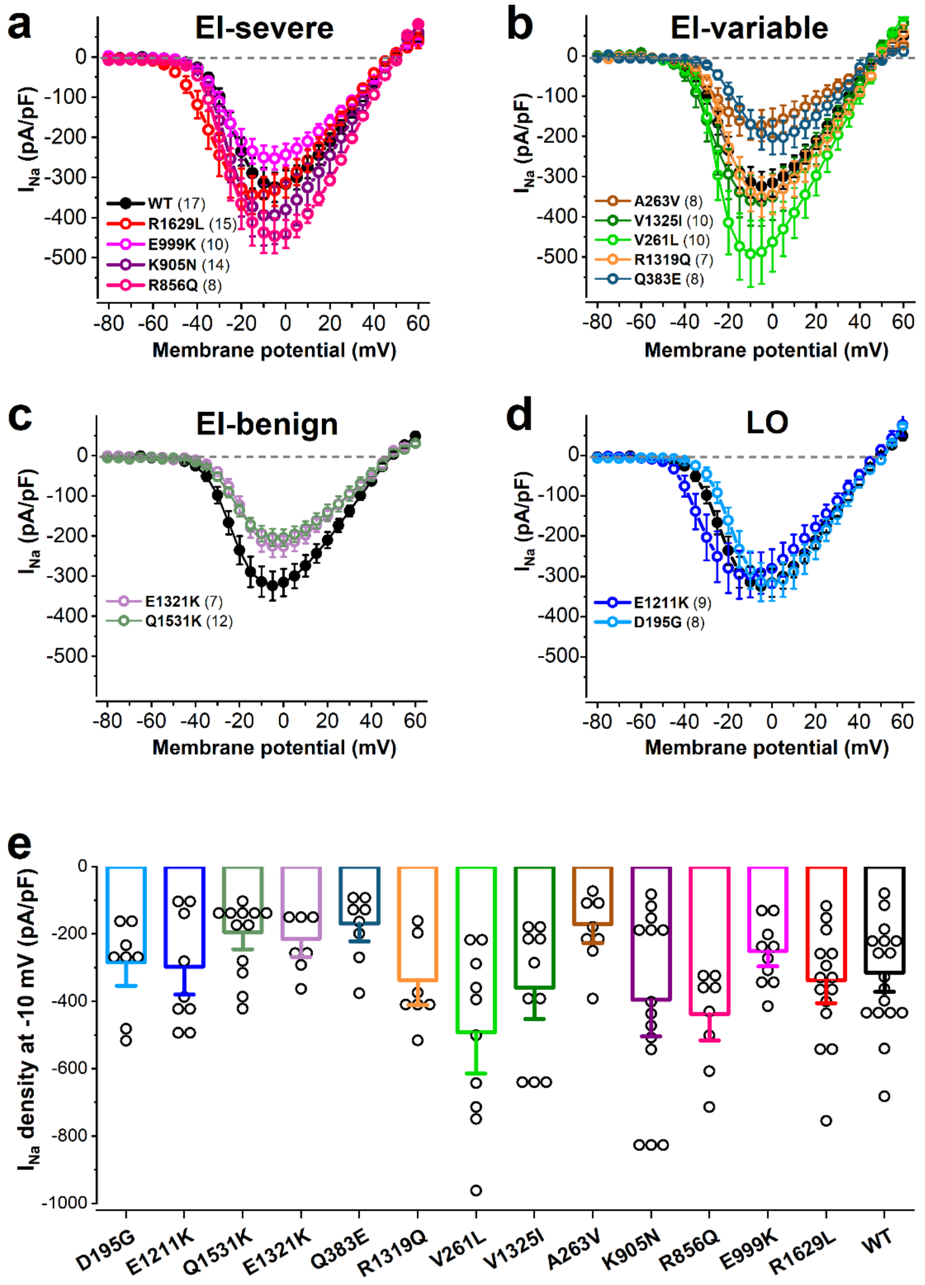

Supplementary Figure 1. Sodium current (I_{Na}) density–voltage relationships in Chinese hamster ovarian (CHO) cells heterologously expressing wild-type (WT, black solid circle) or mutant (colored open circles) $Na_V1.2$ channels associated with early-infantile (EI) severe (**a**), variable (**b**), benign (**c**), or later-onset (LO) (**d**) *SCN2A* epilepsy. **e** Mean I_{Na} densities and data for individual cells (black open circles) recorded at the membrane potential value of −10 mV (WT, black open bar; mutant, colored open bars). Data shown are mean ± SEM; n values, representing the number of independents experiments, are shown in parentheses. The I_{Na} density values of the variants were similar to that of WT (one-way ANOVA, followed by Dunnett's post-hoc test); individual P values are shown in Supplementary Table 3.

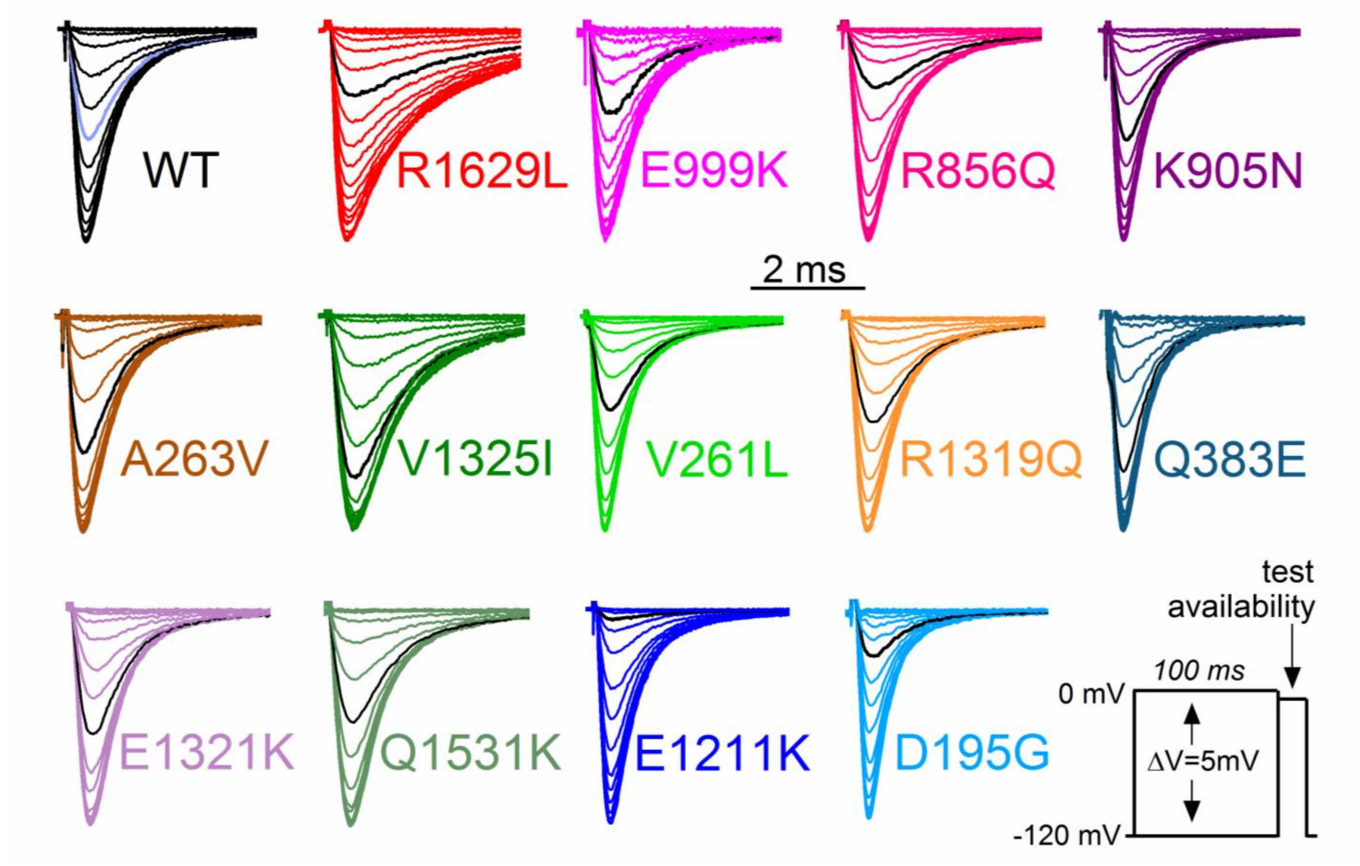

Supplementary Figure 2. Steady-state inactivation of Na_V1.2 channel variants. Representative Na^+ current availability traces, recorded at −5 or −10 mV, immediately after 100-ms pre-pulses to voltages between −120 and 0 mV, applied every 10 s from a holding potential of −120 mV (*inset* voltage protocol).
The membrane potential for half-maximal inactivation ($V_{0.5,inact}$) values and their statistical evaluation are shown in Supplementary Table 3. For each variant, the current trace recorded after the 100-ms pre-pulse at −50 mV is highlighted in light grey (WT, wild-type) or black (mutants). Current traces of individual variants were normalized to the same amplitude; note inset time scale bar. The voltage dependence of inactivation of WT and mutant Na_V1.2 channel variants is shown in Figure 2 (main text).

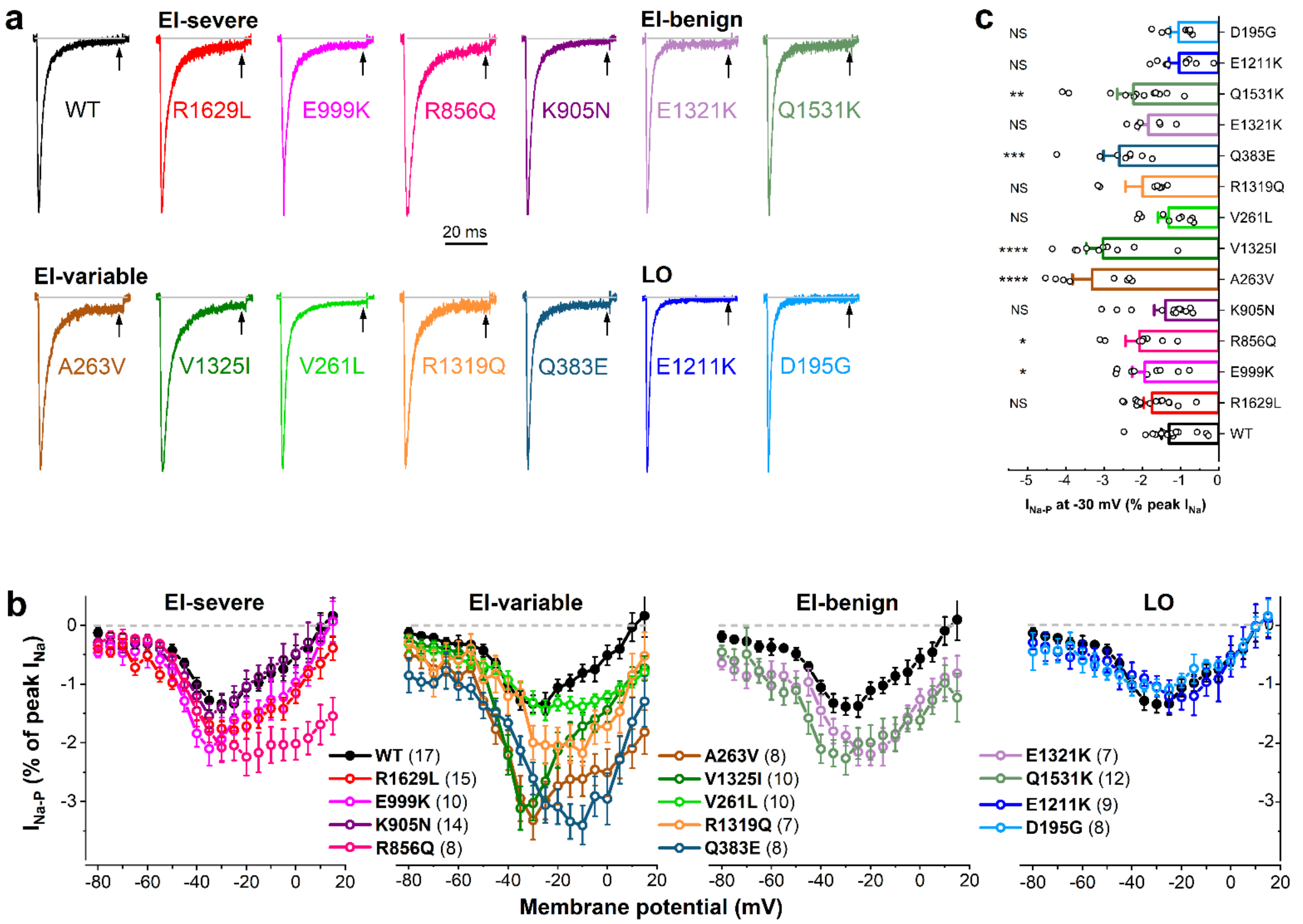

Supplementary Figure 3. Absence and presence of persistent Na$_V$1.2 currents ($I_{Na\text{-}P}$) in Chinese hamster ovarian (CHO) cells heterologously expressing wild-type (WT) or mutant Na$_V$1.2 channels associated with early-infantile (EI) severe/variable/benign, or later-onset (LO) *SCN2A* epilepsy. **a** Representative Na$_V$1.2 traces showing $I_{Na\text{-}P}$ (arrows), 40 ms after the onset of a −30 mV depolarizing voltage step. Current traces of individual variants were normalized to the same amplitude; horizontal grey lines indicate zero current level; horizontal scale time scale bar: 20 ms. **b** Mean $I_{Na\text{-}P}$–voltage relationships, expressed as percentage of peak sodium current (I_{Na}): WT, black solid circle; mutants, colored open circles. Currents were elicited from a holding potential of −120 mV, using 40 ms depolarizing voltage steps of 5 mV increments in the voltage range between −80 and +20 mV, at 0.5 Hz. Data shown are mean ± SEM; n, the number of independent experiments, is shown in parentheses. **c** $I_{Na\text{-}P}$ values, elicited at −30 mV: WT, black open bar; mutant, colored open bars. Data for individual cells are shown in open circles. NS, statistically not significantly different relative to WT. $I_{Na\text{-}P}$ increased over a relatively wide range of membrane potentials in cells expressing E999K, R856Q (EI-consistently severe), A263V, V1325I, Q383E (EI-variable severity), and Q1531K (EI-consistently benign) variants compared with WT, whereas $I_{Na\text{-}P}$ remained unchanged for the other EI and the LO phenotype variants. Data shown are mean ± SEM; the n values are shown in b. $^{*}P < 0.05$ (one-way ANOVA, followed by Dunnett's post-hoc test); see the P values in Supplementary Table 3.

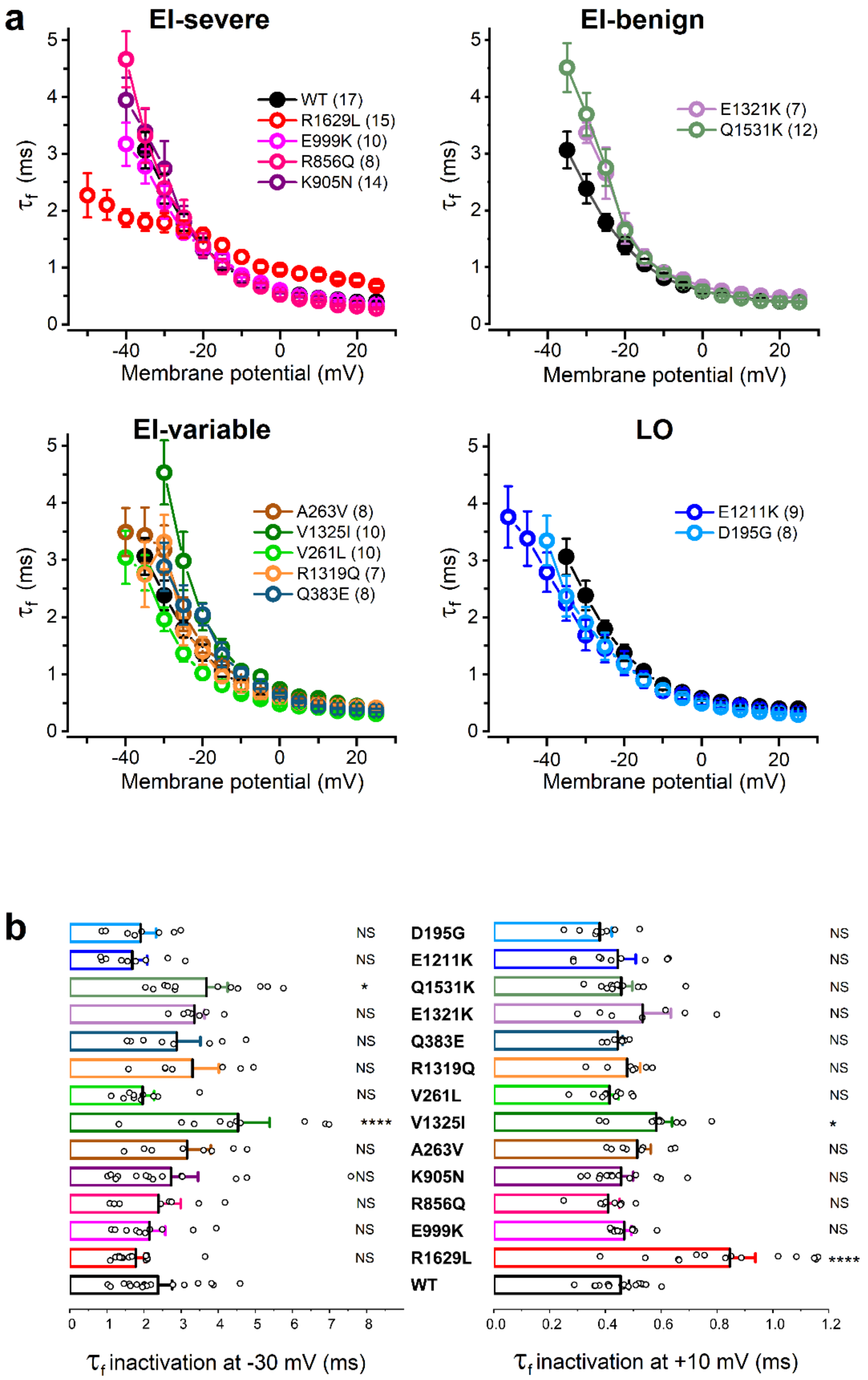

Supplementary Figure 4. Time constants of inactivation for wild-type (WT) and $Na_V1.2$ channel variants associated with early-infantile (EI) severe/variable/benign, or later-onset (LO) *SCN2A* epilepsy. **a** Fast inactivation time constants (τ_f) (WT, black solid circle; mutants, colored open circles), obtained by analysing the decay of individual I_{Na} traces elicited at various depolarizing voltages using a double-exponential equation: $y = A_f e^{-t/\tau_f} + A_s e^{-t/\tau_s}$, where t is time, A_f and A_s are the fractions of the fast and slow inactivation components, and τ_f and τ_s are the time constants of the fast and slow inactivating components, respectively. Mean τ_f values were plotted against the membrane potential; n values, the number of independent experiments, are shown in parentheses. **b** Mean τ_f values for traces elicited at −30 and +10 mV: WT, black open bar; mutant, colored open bars. Data for individual cells are shown in open circles. Data shown are mean ± SEM; $^*P < 0.05$ (one-way ANOVA, followed by Dunnett's post-hoc test); NS, statistically not significantly different relative to WT; see the P values in Supplementary Table 3.

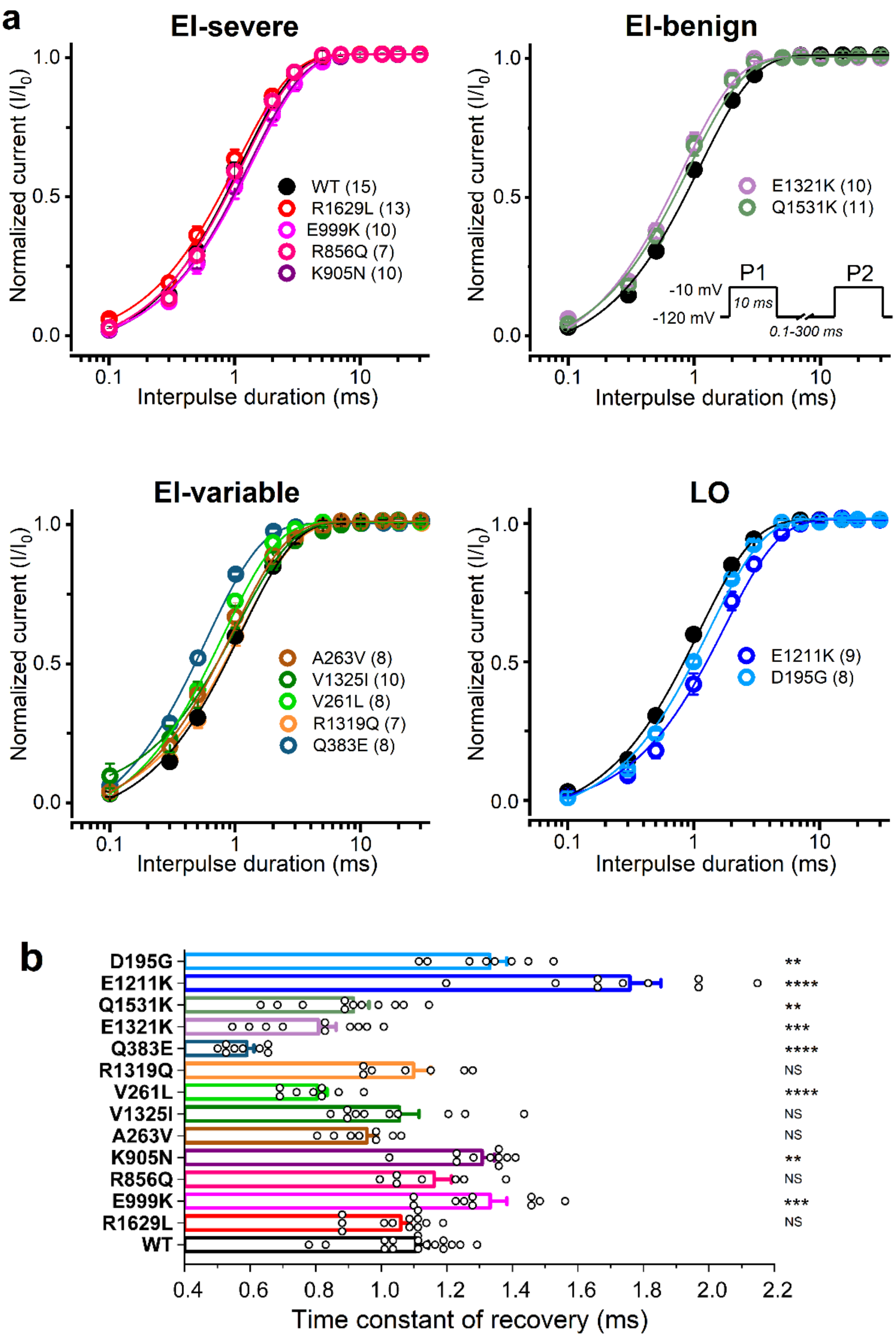

Supplementary Figure 5. Recovery from inactivation of wild-type (WT), early-infantile (EI) severe/variable/benign, or later-onset (LO) $Na_V1.2$ channel variants. **a** Recovery was assessed using a paired-pulse voltage protocol detailed in the Methods, from a holding potential of –120 mV (*inset protocol*); WT, black solid circle; mutants, colored open circles; n, the number of independent experiments, is shown in parentheses. **b** Mean τ values of recovery: WT, black open bar; mutant, colored open bars. Data for individual cells are shown in open circles. Relative to WT channels, the V261L, Q383E, E1321K, and Q1531K variants recovered faster, corresponding to gain-of function (GoF), whereas E999K, K905N, E1211K, and D195G showed slower recoveries, consistent with loss-of-function (LoF); the recoveries of R1629L, R856Q, A263V, V1325I, and R1329Q were unchanged. Data shown are mean ± SEM; $^{*}P < 0.05$ (one-way ANOVA, followed by Dunnett's post-hoc test); NS, statistically not significantly different relative to WT; see the P values in Supplementary Table 3.

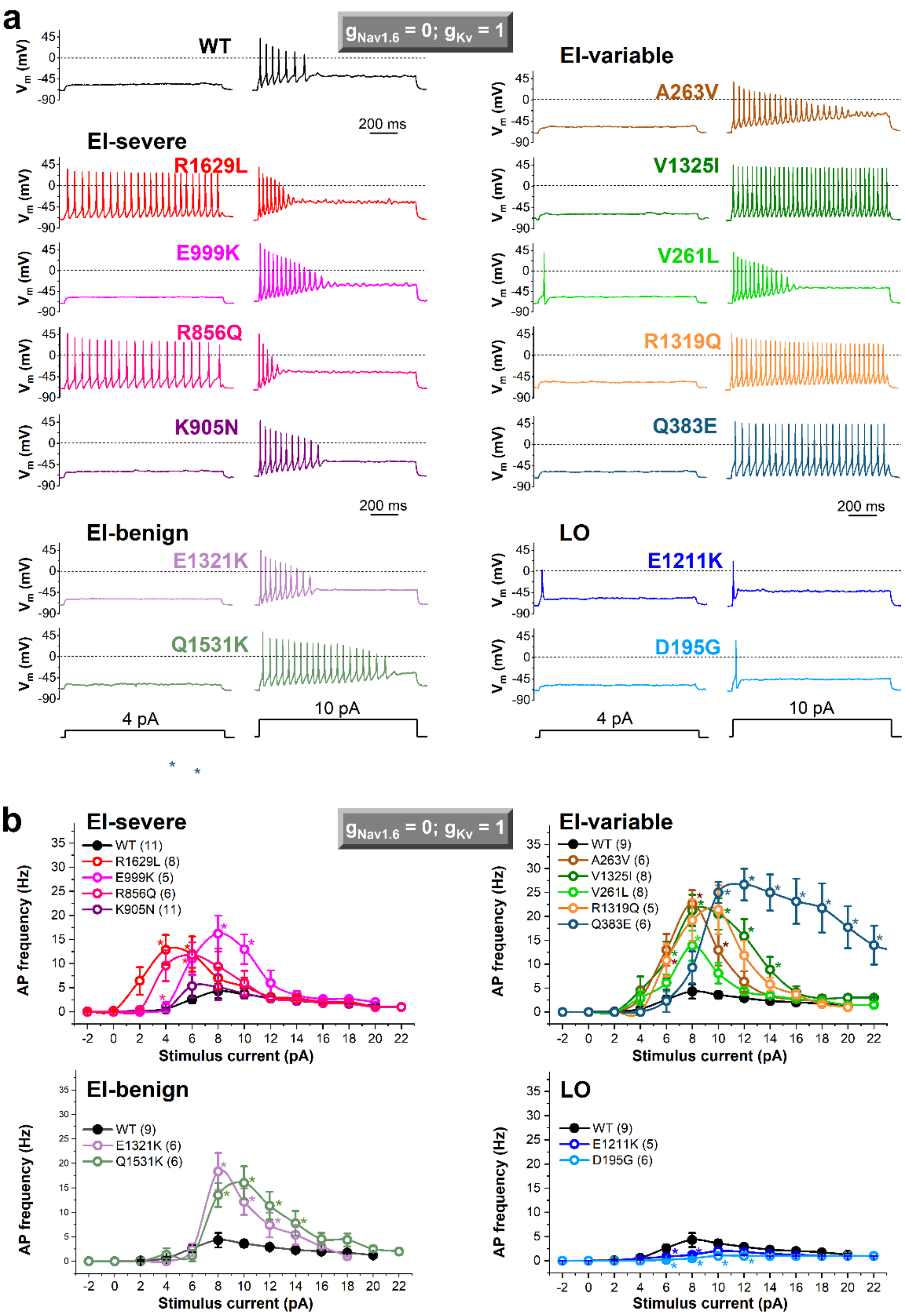

Supplementary Figure 6. Dynamic action potential clamp (DAPC) experiments implementing external $Na_V1.2$ currents from Chinese hamster ovary (CHO) cells expressing wild-type (WT), early-infantile (EI) severe/variable/benign, or later-onset (LO) $Na_V1.2$ variants. In the axon initial segment compartment model, the $Na_V1.6$ conductance ($gNa_V1.6$) was set to zero, whereas the potassium channel conductance (gKv) was set to 100% ($gNa_V1.6$ = 0, gK_V = 1, respectively). See also the effect of a gKv setting of 2 in Figure 3 (main text). **a** Representative examples of action potential firing in response to 4 and 10 pA step current stimuli. **b** Input–output relationships. Note the overall lower AP firing frequencies compared to data shown in Figure 3. Data shown are mean ± SEM; Two-way ANOVA, followed by Dunnett's post-hoc test, was used to compare the action potential firing frequencies elicited by step stimuli in the presence of $Na_V1.2$ variants (WT, black solid circle; mutants, colored open circles); asterisks indicate $P < 0.05$; n values, the number of independent experiments, are shown in parentheses.

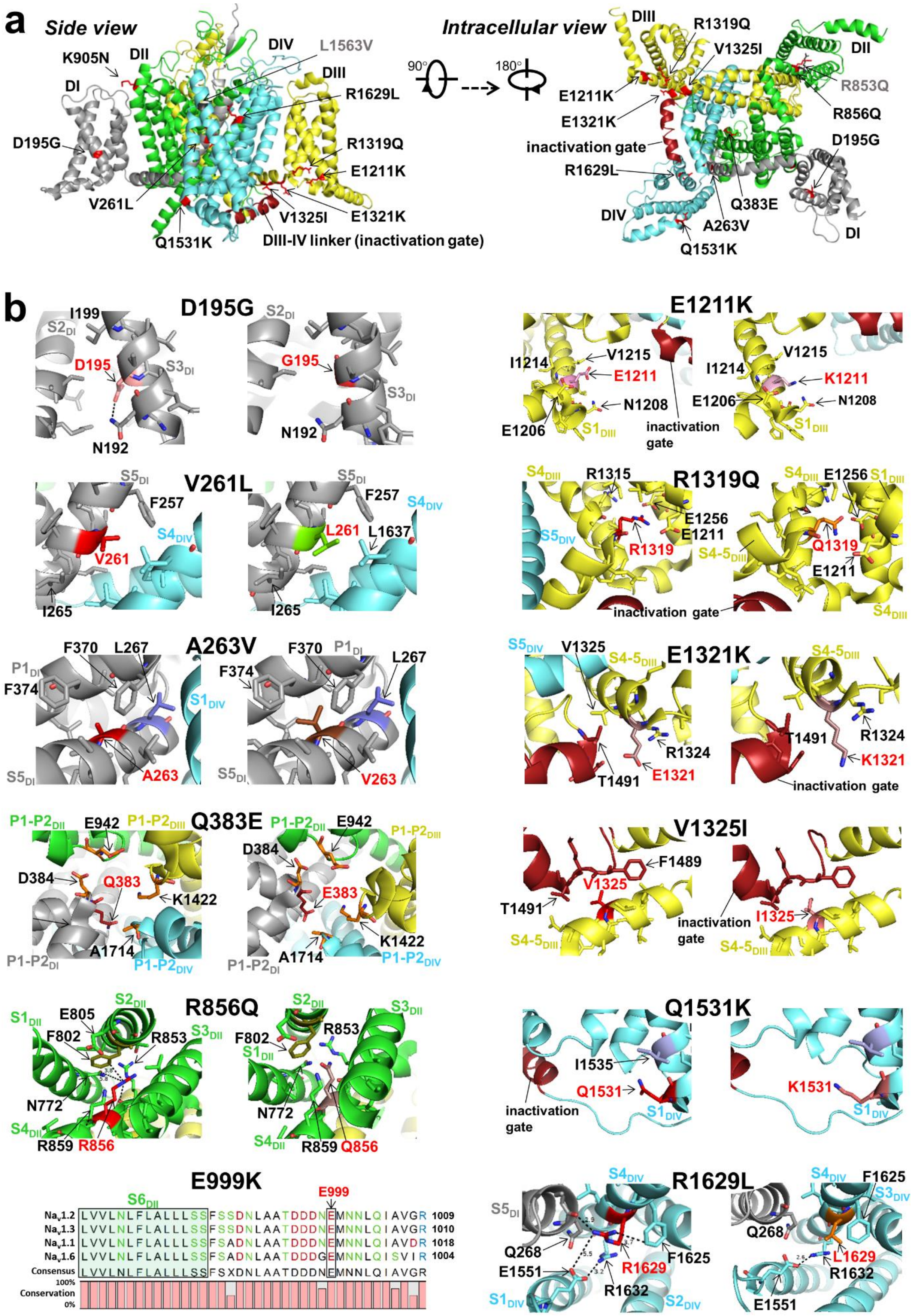

Supplementary Figure 7. 3D structures of the functionally studied Nav1.2 channel variants, shown as cartoon (PDB accession no. 6J8E)[1]. The variants are localized in channel domains or segments associated

with gating or ion permeation (see details in the main text)[2]. **a** Side and intracellular views of the wild-type channel highlighting the residues affected by missense mutations (red sticks). The four domains, DI-IV, are colour-coded; modeling was performed in PyMOL (Schrödinger LLC, New York, USA). **b** Zoomed-in views of structures in Nav1.2, highlighting residues before (left) and after (right) in silico mutagenesis. All residues within 5Å distance from the mutated residue are shown in stick representation (blue: nitrogen, red: oxygen). **D195G** (associated with later-onset (LO) phenotype): negatively charged Asp residue mutated to uncharged Gly residue in segment 5 of domain I ($S5_{DI}$); the polar interaction between D195 and N192, shown in wild-type channel, cannot be formed in the mutant variant. **V261L** (early-infantile (EI)-variable): hydrophobic Val residue mutated to hydrophobic Leu residue in $S5_{DI}$; the longer side chain of Leu may affect the hydrophobic interactions within DI. **A263V** (EI-variable): hydrophobic Ala residue mutated to hydrophobic Val residue in $S5_{DI}$; it is likely that this mutation affects hydrophobic side chain interactions within DI. **Q383E** (EI-variable): polar uncharged Gln residue mutated to negative Glu residue in P1-P2 of DI; Gln383 is adjacent to the key Glu384 residue in the DEKA selectivity filter. Within the DEKA motif, the positively charged Lys and the carboxylate from Glu are responsible for maintaining an ionic permeability ratio of 0.03:0.075 for K^+ over Na^+ [3]. Monte Carlo simulations suggest that the selectivity filter of the wild-type $Na_V1.2$ channel has an electrostatic balance of 2 positive (K1422 and the Na^+ ion) and 2 negative (D384 and E942)[4]. In the wild-type channel, Q383 contributes to stabilizing the selectivity filter by donating a H-bond to the backbone carbonyl of L380 (and/or R379). It has been suggested that the Q383E channel may be Ca^{2+} permeable because E383 provides an additional negative charge to the DEKA motif (3 negative: E942, D384, and E383), which can be balanced with 3 positive charges carried by K1422 and Ca^{2+} [4]. However, this hypothesis has not yet been tested experimentally. **R856Q** (EI-severe): Positive Arg mutated to Gln (with polar uncharged side chain) neutralizing mutation in $S4_{DII}$. In the wild-type channel, the gating charge-carrying Arg residues in segment 4 (S4) and several negative residues in S1 and S2 of the voltage sensor domain are involved in channel state-dependent interactions, resulting in a network of ionic and hydrogen-bonding interactions[5]. In the R856Q variant, some of the above interactions, including the polar contact between positions R856 and R853, and the cation-π interaction[6] between the sidechain of R856 and the aromatic sidechain of F802 are disrupted. **E999K** (EI-severe): Negative Glu residue mutated to positive Lys in the intracellular DII-DIII linker. The structure of DII-DIII linker is currently unresolved. Sequence alignment of the proximal DII-DIII linker of $Na_V1.2$, $Na_V1.3$, $Na_V1.1$, and $Na_V1.6$ channels, performed with CLC Sequence Viewer 7.7 (QIAGEN Aarhus, Denmark). The residue colours correspond to polarity. The green-shaded boxed area highlights the intracellular terminal of $S6_{DII}$. Note that the E999 residue of $Na_V1.2$ is conserved across the brain sodium channels. Various physiological roles have been attributed to the DII-DIII linker, including the regulation of clustering of sodium channels at the axonal initial segment[7], ankyrin binding[8], and gating[9]. **E1211K** (LO): Negative Glu residue mutated to positive Lys in $S1_{DIII}$; E1211 is highly conserved in across voltage-gated sodium and calcium channels. E1211 forms polar contacts with V1215 and I1214. In the mutant channel, additional interactions may be formed between K1211 and E1206, and/or K1211 and N1208. **R1319Q** (EI-variable): Positive Arg residue mutated to polar uncharged Gln residue in $S4_{DIII}$. This mutation is likely to affect the movement of the voltage sensor[10]. **E1321K** (EI-benign): Negative Glu residue mutated to positive Lys in the intracellular S4-S5 linker in DIII ($S4\text{-}5_{DIII}$). Mutations in the in the S4–S5 linker are likely to disrupt the various coupling interactions between the voltage sensor and the pore[11]. **V1325I** (EI-variable): hydrophobic Val residue mutated to hydrophobic Ile residue in $S4\text{-}5_{DIII}$. See the role of S4–S5 linker above (E1321K). Note the proximity of residue F1489, a key IFM hydrophobic sequence motif involved in fast inactivation[2]. **Q1531K** (EI-benign): Glu residue with polar uncharged side chain mutated

to positive Lys in $S1_{DIV}$. Q1531 of $Na_v1.2$ is a conserved residue across human sodium channels. **R1629L** (EI-severe): Positive Arg residue mutated to hydrophobic Leu residue in $S4_{DIV}$. In the wild-type channel, the R1 to R4 gating charges reside above the hydrophobic constriction site, when VSD4 is in an activated conformation. R1629 forms polar interactions with Q268 ($S5_{DI}$) and E1551 ($S4D_{IV}$), and cation-π interaction with the aromatic sidechain of F1625 ($S1D_{IV}$)[6]. These interactions cannot be formed in the R1629L variant. Dashed lines indicate polar interactions with a number denoting the distance between atoms in Å.

SUPPLEMENTARY TABLES

Supplementary Table 1. Allocation to early-infantile, late-onset and intellectual disability/autism spectrum disorder (ID/ASD) phenotypic groups. Phenotypic groups were defined according to age of seizure onset, and additional clinical features if seizure onset was at ≥ age three months.

Seizure onset	Variants (N)	Individuals (N)	Comments
Early-infantile phenotypic group (N=144)			
< 3 months	26	75	Variants: R36C, N212D, M252V, V261L, V261M, A263V, D343G, Q383E, V523L, R856Q, G882E, K908E, S987I, E999K, R1319Q, E1321K, V1325I, S1336Y, M1338T, Q1531K, L1563V, R1626Q, R1629H, F1651C, R1882G, R1882Q
3-24 months if:<br><br>-no epileptic spasms AND<br><br>-normal pre-seizure development (or unknown pre-seizure development and normal/near normal outcome)	10	36	Variants: R36G, V208E, M252V, D343G, Q383E, R1319Q, E1321K, V1325I, L1563V, Y1589C<br><br>Phenotypic notes:<br><br>Development prior to seizure onset normal in 19, and unknown in 17 (development at last review normal in 15, mildly delayed in 2).<br><br>Two individuals with mildly delayed developmental outcome had seizure onset at ages 3 and 6 months, and one also had episodic ataxia. These individuals had Q383E and D343 variants.<br><br>Seizure onset between age 3-24 months seen in at least one individual with 10/30 variants associated with early-infantile phenotype. 8/10 also seen in at least one individual with seizure onset <3 months (including D343G and Q383E) and 2/10 (V208E, Y1589C) with at least one individual with seizure onset at age 3 months.
Unknown age if:<br><br>-presenting epilepsy syndrome S(F)NIS, EIEE or EIMFS	5	28	Variants: R223Q, M252V K908E, E999K, L1330F<br><br>Phenotypic notes:<br><br>S(F)NIS in 27/28, EIEE in 1/28
No seizures if:<br><br>-unaffected member of a family with early-onset phenotype who has the *SCN2A* variant (incomplete	4	5	Variants: M252V, A263V, R1319Q, Y1589C<br><br>Phenotypic notes: all four variants not present in gnomAD.

penetrance/variable expressivity)			
Later-onset phenotypic group (N=29)			
≥ 3 months (unless meet criteria above)	7	27	Variants: D195G, R220Q, K503fs*, R853Q, E1211K, L1342, R1435*<br><br>Phenotypic notes:<br><br>Seizure onset between 3-24 months in 24, 20/24 having epileptic spasms. 17/24 had delayed development prior to seizure onset; pre-seizure development was normal in 1 and unknown in 6 (all 7 had epileptic spasms).<br><br>Seizure onset >24 months in three
Unknown age if:<br><br>-presenting epilepsy syndrome WS, LGS or MAE	1	2	Variants: R853Q<br><br>One with WS, one with LGS
ID/ASD without epilepsy phenotypic group (N=6)			
No seizures if:<br><br>-has ID/ASD	3	6	Variants: K503fs*, R937C, R1435*<br><br>Ages at last review: 3 years, 7 years, 18 years, unknown in three

Supplementary Table 2. Subdivision of early-infantile phenotypic groups in *SCN2A*-related disorders.

Early-infantile phenotypic subdivisions	Seizures after age two years	Developmental outcome	Other neurologic symptoms
Benign	-	Normal	-
Intermediate	+/-	Normal-moderately impaired	+/-<br>(may include episodic ataxia, hypotonia)
Severe	+/-	Severe-profoundly impaired	+<br>(may include abnormal tone, movement disorders, microcephaly)

Supplementary Table 3. Biophysical characteristics of the $Na_V1.2$ channel variants in VC experiments.

Variant	Biophysical property					
	I_{Na} density at –10 mV (pA/pF)	$V_{0.5,act}$ (mV)	$V_{0.5,inact}$ (mV)	$I_{Na\text{-}P}$ at –30 mV (% of peak)	τ_f inactivation at –30 mV (ms)	τ recovery (ms)
**WT**	313.8 ± 38	–17.75 ± 0.45	–49.21 ± 0.50	1.30 ± 0.13	2.38 ± 0.25	1.10 ± 0.03
n	17	17	16	17	17	15
P value	–	–	–	–	–	–
**R1629L**	342.9 ± 44 ^{NS}	–24.44 ± 0.53****	–54.97 ± 0.32****	1.76 ± 0.14 ^{NS}	1.78 ± 0.17 ^{NS}	1.06 ± 0.03 ^{NS}
n	15	15	15	15	15	13
P value	0.999	<0.0001	<0.0001	0.141	0.443	0.998
**E999K**	250.4 ± 30 ^{NS}	–19.60 ± 0.40*	–53.06 ± 0.74****	1.94 ± 0.22*	2.15 ± 0.29 ^{NS}	1.37 ± 0.05***
n	10	10	9	10	10	10
P value	0.988	0.029	<0.0001	0.461	0.972	0.0001
**K905N**	394.0 ± 75 ^{NS}	–18.72 ± 0.42^{NS}	–52.99 ± 0.65****	1.40 ± 0.19 ^{NS}	2.73 ± 0.49 ^{NS}	1.31 ± 0.04**
n	14	14	14	14	14	10
P value	0.877	0.539	<0.0001	0.985	0.848	0.0054
**R856Q**	437.1 ± 51 ^{NS}	–20.98 ± 0.42****	–55.90 ± 0.42****	2.07 ± 0.25*	2.39 ± 0.39 ^{NS}	1.16 ± 0.05 ^{NS}
n	8	8	8	8	8	7
P value	0.612	<0.0001	<0.0001	0.021	>0.999	0.988
**A263V**	170.9 ± 37 ^{NS}	–20.25 ± 0.38**	–46.57 ± 0.53**	3.31 ± 0.36****	3.17 ± 0.43 ^{NS}	0.95 ± 0.03 ^{NS}
n	8	8	8	8	8	8
P value	0.411	0.0023	0.004	<0.0001	0.546	0.165
**V1325I**	359.2 ± 62 ^{NS}	–23.08 ± 0.40****	–43.62 ± 0.33****	3.02 ± 0.29****	4.53 ± 0.56****	1.05 ± 0.06 ^{NS}
n	10	10	10	10	10	10
P value	0.999	<0.0001	<0.0001	<0.0001	<0.0001	0.990
**V261L**	491.6 ± 82 ^{NS}	–21.26 ± 0.29****	–52.24 ± 0.59***	1.31 ± 0.19 ^{NS}	1.96 ± 0.21 ^{NS}	0.80 ± 0.03****
n	10	10	10	10	10	8
P value	0.103	<0.0001	0.0001	>0.999	0.959	<0.0001
**R1319Q**	337.5 ± 48 ^{NS}	–17.97 ± 0.41^{NS}	–49.12 ± 0.54 ^{NS}	1.99 ± 0.29 ^{NS}	3.31 ± 0.47 ^{NS}	1.09 ± 0.05 ^{NS}
n	7	7	7	7	7	7
P value	0.999	0.999	0.999	0.179	0.401	0.999
**Q383E**	169.7 ± 35 ^{NS}	–12.26 ± 0.55****	–42.06 ± 0.38****	2.60 ± 0.25***	2.88 ± 0.42 ^{NS}	0.58 ± 0.02****
n	8	7	7	8	8	8
P value	0.400	<0.0001	<0.0001	0.0006	0.928	<0.0001
**E1321K**	213.4 ± 36 ^{NS}	–17.65 ± 0.47 ^{NS}	–45.85 ± 0.53****	1.84± 0.17 ^{NS}	3.36 ± 0.18 ^{NS}	0.80 ± 0.05***
n	7	7	8	7	7	7
P value	0.873	0.999	<0.0001	0.524	0.339	0.0001
**Q1531K**	194.0 ± 33 ^{NS}	–16.67 ± 0.39 ^{NS}	–47.01 ± 0.40**	2.23 ± 0.28**	3.69 ± 0.38*	0.91 ± 0.04**
n	12	12	12	12	12	12
P value	0.476	0.451	0.007	0.008	0.022	0.009
**E1211K**	296.3 ± 55 ^{NS}	–25.44 ± 0.59****	–72.52 ± 0.38****	1.03 ± 0.18 ^{NS}	1.68 ± 0.27 ^{NS}	1.76 ± 0.09****
n	9	9	9	9	9	9
P value	0.999	<0.0001	<0.0001	0.966	0.642	<0.0001
**D195G**	283.7 ± 46 ^{NS}	–10.25 ± 0.34****	–61.20 ± 0.47****	1.05 ± 0.14 ^{NS}	1.91 ± 0.28 ^{NS}	1.33 ± 0.05**
n	8	8	8	8	8	8
P value	0.995	<0.0001	<0.0001	0.984	0.949	0.004

Statistically significant differences between the wild-type (WT) and mutant channels were determined using one-way ANOVA, followed by Dunnett's post-hoc test ($^{*}P < 0.05$, $^{**}P < 0.01$, $^{***}P < 0.001$, and $^{****}P < 0.0001$); NS, statistically not significant difference compared to WT. Data are represented as mean ± SEM. Abbreviations: I_{Na}, sodium current; $I_{Na\text{-}P}$, persistent sodium current; n, number of independent experiments; τ_f, fast time constant (of the inactivation); τ recovery, time constant of recovery from fast inactivation; $V_{0.5,act}$, membrane potential for half-maximal activation;$V_{0.5,inact}$, membrane potential for half-maximal inactivation; VC, voltage clamp.

Supplementary Table 4. Clinico-electrophysiological severity score of $Na_v1.2$ variants in the early-infantile phenotypic group.

Early-infantile variant		Electro-phys. alter-ation yes/no	Δ $V_{0.5,act}$ (mV)	$V_{0.5,act}$ score	Δ $V_{0.5,inact}$ (mV)	$V_{0.5,inact}$ score	Δ $V_{0.5}$ with neutral-ising effect	$V_{0.5}$ score (with neutral -ising effect)	Δ $I_{Na\text{-}P}$ (%)	$I_{Na\text{-}P}$ score	#Δ recovery	Recovery score	Other strong effects (i.e., recovery or $I_{Na\text{-}P}$) yes/no	Strongest effect of all mutations yes/no	More than three features affected yes/no	CESSNa+ score	CESSNa+ score (with 'neutral-ising' effect)
Early-infantile -severe	R1629L	1	-6.69	3	-5.75	3	-0.94	0	0.46	1	-0.05	0	0	1	1	9	3
	E999K	1	-1.83	1	-3.84	2	-2.01	1	0.63	1	0.27	2	0	0	1	5	3
	R856Q	1	-3.21z	2	-6.69	3	-3.48	2	0.77	2	0.06	0	0	0	1	7	4
	K905N	1	-0.97	0	-3.78	2	-2.81	2	0.09	0	0.2	2	0	0	0	3	1
	R1882Q*	1	-6.02	3	4.37	2	N/A	N/A	1.74	3	0.02	0	1	0	1	8	N/A
Early-infantile -variable	A263V	1	-2.5	2	2.61	2	N/A	N/A	2.01	3	-0.15	2	1	1	1	8	N/A
	V1325I	1	-5.33	3	5.59	3	N/A	N/A	1.72	3	-0.04	0	1	0	1	9	N/A
	V261L	1	-3.51	2	-3.03	2	-0.48	2	0.01	0	-0.3	2	0	0	1	6	4
	R1319Q	1	-0.22	0	0.1	0	N/A	N/A	0.69	3	-0.01	0	1	0	0	2	N/A
	Q383E	1	5.49	3	-7.17	3	N/A	N/A	1.3	2	-0.52	3	1	2	1	11	N/A
Early-infantile -benign	E1321K	1	-0.1	0	3.34	2	N/A	N/A	0.54	1	-0.3	2	0	0	1	4	N/A
	Q1531K	1	1.08	0	1.96	1	0.88	0	0.93	3	-0.19	1	1	0	1	4	3
	L1563V*	1	-1.67	1	2.52	2	N/A	N/A	0.05	0	-0.4	3	1	0	1	6	N/A

mild intermediate strong strongest effect of all mutations

Early-infantile $Na_v1.2$ variants were allocated into benign, variable, and severe phenotypic groups. The clinico-electrophysiological severity score of $Na_v1.2$ variants (CESSNa+ score) was calculated as described according to Lauxmann et al[12] (see Methods). Briefly, the maximum effect of all 13 variants was determined relative to control for each assessed biophysical property, and divided in three thirds corresponding to large, medium, and small changes, associated with high (3), medium (2) and low (1) scores. Additional points were scored for the maximal (strongest) change of a selected biophysical property (e.g., persistent sodium current and recovery from fast inactivation) and if multiple (at least three) biophysical properties were affected by a mutation for a given variant. Opposing effect, such as the shifts of the $V_{0.5,act}$ and $V_{0.5,inact}$ in the same direction were considered as 'neutralising'. A high CESSNa+ score was correlated with pronounced severity[12]. Two sets of CESSNa+ scores were generated, by considering or omitting the neutralising effect. . Mean CESSNa+ scores in the benign, variable, and severe phenotypic groups were compared using one-way ANOVA and the P value determined. Mean CESSNa+ scores in the early-infantile-severe, early-infantile-variable, and early-infantile-benign groups were statistically not significantly different (P = 0.46; one-way ANOVA). These results suggest that, for this relatively small dataset, the CESSNa+ score cannot differentiate early-infantile-severe or early-infantile-variable groups from early-infantile-benign. CESSNa+ scores should be regarded as an indication of variant severity rather than an unequivocal prediction of variant severity[12].

Abbreviations: $V_{0.5,act}$, half-point of activation; $V_{0.5,inact}$, half-point of inactivation; $I_{Na\text{-}P}$, persistent current; N/A, not applicable; *, Early-infantile variants studied previously[13]; #, negative and positive values differentiate between variants with faster and slower recoveries vs wild-type, respectively.

Supplementary Table 5. Action potential firing activity during step current stimulation in dynamic action potential clamp experiments using $Na_V1.2$ variant current and virtual conductance settings of $gNa_V1.6$ = $0/gK_V$ = 2.

Variant	Effects of step current (I_{st}) magnitude on firing frequency (Hz)					
	I_{st} = 2 nA	I_{st} = 4 nA	I_{st} = 8 nA	I_{st} = 10 nA	I_{st} = 12 nA	I_{st} = 14 nA
**WT**	NF	0.57 ± 0.29	4.42 ± 1.19	10.1 ± 2.49	9.41 ± 2.08	5.42 ± 1.80
n	9	9	9	9	9	9
P value	–	–	–	–	–	–
**R1629L**	9.00 ± 2.97****	13.4 ± 3.25***	19.6 ± 3.13**	13.3 ± 3.39^{NS}	9.83 ± 3.92^{NS}	5.50 ± 1.55^{NS}
n	7	7	7	7	7	7
P value	<0.0001	0.0002	0.00023	0.767	0.758	>0.334
**E999K**	NF^{NS}	2.50 ± 2.50^{NS}	17.0 ± 3.4**	19.5 ± 3.74***	21.8 ± 4.57***	16.5 ± 4.42**
n	5	5	5	5	5	5
P value	>0.950	0.629	0.0025	0.0009	0.0008	0.006
**R856Q**	NF^{NS}	10.0 ± 3.42**	15.6 ± 3.1**	14.8 ± 2.67^{NS}	11.0 ± 2.12^{NS}	10.6 ± 2.03^{NS}
n	6	6	6	6	6	6
P value	0.938	0.0035	0.008	0.054	0.939	0.375
**K905N**	NF^{NS}	0.22 ± 0.22^{NS}	6.77± 2.78^{NS}	6.88 ± 1.88^{NS}	4.88 ± 2.05^{NS}	3.44 ± 1.12^{NS}
n	9	9	9	9	9	9
P value	0.991	0.737	0.920	0.992	0.899	0.992
**A263V**	NF^{NS}	1.40 ± 0.87^{NS}	28.6 ± 2.60****	32.4 ± 2.88****	25.8 ± 3.49****	15.0 ± 3.42*
n	6	6	6	6	6	6
P value	>0.999	0.997	<0.0001	<0.0001	<0.0001	0.034
**V1325I**	3.71 ± 2.48^{NS}	10.1 ± 3.88*	23.3 ± 4.43****	26.4± 4.01****	22.9 ± 4.18***	17.3 ± 3.75**
n	7	7	7	7	7	7
P value	0.740	0.024	<0.0001	<0.0001	0.0005	0.0028
**V261L**	0.75 ± 0.75^{NS}	4.12 ± 2.20^{NS}	17.5 ± 2.46***	21.8 ± 2.71**	19.9 ± 3.40**	10.1 ± 2.59 ^{NS}
n	8	8	8	8	8	8
P value	0.997	0.746	0.0004	0.0023	0.007	0.497
**R1319Q**	0.60 ± 0.60^{NS}	1.40 ± 0.97^{NS}	25.0 ± 5.87****	26.2 ± 4.62****	18.4 ± 5.42^{NS}	7.60 ± 3.55 ^{NS}
n	6	6	6	6	6	6
P value	0.997	0.997	<0.0001	<0.0001	0.053	0.968
**Q383E**	NF^{NS}	NF^{NS}	10.8 ± 6.42^{NS}	25.8 ± 2.81****	32.8 ± 2.47****	35.2 ± 3.62****
n	6	6	6	6	6	6
P value	>0.999	0.998	0.277	<0.0001	<0.0001	<0.0001
**E1321K**	NF^{NS}	0.25 ± 0.20^{NS}	21.3 ± 3.41****	25.3± 4.58****	17.3 ± 4.20**	11.5 ± 3.46*
n	6	6	6	6	6	6
P value	>0.999	0.989	<0.0001	<0.0001	0.0071	0.044
**Q1531K**	NF^{NS}	NF^{NS}	13.3 ± 3.40**	21.5 ± 3.96****	23.0 ± 3.95****	18.6 ± 3.61****
n	6	6	6	6	6	6
P value	>0.999	0.968	0.0022	<0.0001	<0.0001	<0.0001
**E1211K**	NF^{NS}	0.36 ± 0.2^{NS}	0.89 ± 0.33*	3.00 ± 0.94****	6.00 ± 2.00 ^{NS}	6.33 ± 1.33^{NS}
n	5	5	5	5	5	5
P value	>0.999	0.987	0.043	<0.0001	0.0526	0.731
**D195G**	NF^{NS}	NF^{NS}	0.16 ± 0.16**	0.83 ± 0.30****	1.16 ± 0.16****	1.16 ± 0.16^{NS}
n	6	6	6	6	6	6
P value	>0.999	0.899	0.0074	<0.0001	<0.0001	0.314

Statistically significant differences between the wild-type (WT) and mutant channels were determined using two-way ANOVA, followed by Dunnett's post-hoc test ($^{*}P < 0.05$, $^{**}P < 0.01$, $^{***}P < 0.001$, and $^{****}P < 0.0001$). Data are mean ±SEM. Abbreviations: n, number of experiments; NF, no firing; NS, statistically not significant difference compared to WT.

Supplementary Table 6. Action potential characteristics of the axon initial segment neuronal model incorporating wild-type or mutant $Na_V1.2$ channels in dynamic action potential clamp experiments.

**Variant**	**Rheobase (pA)**	**Threshold (pA)**	**Upstroke velocity (dV/dt)**	**Amplitude (mV)**	**Width (ms)**	**Decay time (ms)**
**WT**	6.34 ± 0.61	–36.60 ± 1.12	204.3 ± 4.1	111.3 ± 1.6	1.49 ± 0.04	2.37 ± 0.11
n	9	9	9	9	9	9
P value	-	-	-	-	-	-
**R1629L**	2.00 ± 0.62****	–43.74 ± 2.85*	181.2 ± 8.6*	103.6 ± 4.1^{NS}	3.13 ± 0.21****	5.75 ± 0.64****
n	7	7	7	7	7	7
P value	<0.0001	0.012	0.011	0.075	<0.0001	<0.0001
**E999K**	5.20 ± 0.48^{NS}	–38.89 ± 1.77^{NS}	211.3 ± 2.0^{NS}	113.9 ± 1.1^{NS}	1.50 ± 0.03^{NS}	2.54 ± 0.11^{NS}
n	5	5	5	5	5	5
P value	0.392	0.785	0.812	0.887	>0.999	0.987
**R856Q**	4.66 ± 0.42^{NS}	–42.11 ± 1.59^{NS}	205.0 ± 4.5^{NS}	117.7 ± 1.6^{NS}	1.46 ± 0.04^{NS}	2.63 ± 0.07^{NS}
n	6	6	6	6	6	6
P value	0.082	0.087	0.999	0.203	0.998	0.933
**K905N**	5.77 ± 0.22^{NS}	–36.63 ± 0.83^{NS}	199.6 ± 4.4^{NS}	114.2 ± 1.9^{NS}	1.55 ± 0.03^{NS}	2.77 ± 0.12^{NS}
n	9	9	9	9	9	9
P value	0.798	>0.999	0.905	0.754	0.970	0.687
**A263V**	5.60 ± 0.74^{NS}	–36.00 ± 1.45^{NS}	193.4 ± 8.6^{NS}	112.2 ± 0.8^{NS}	1.91 ± 0.07***	3.31 ± 0.17*
n	6	6	6	6	6	6
P value	0.917	0.998	0.830	0.993	0.0004	0.011
**V1325I**	4.57 ± 0.71^{NS}	–38.88 ± 1.78^{NS}	197.8 ± 3.2^{NS}	111.1 ± 2.0^{NS}	1.88 ± 0.10***	3.37 ± 0.25**
n	7	7	7	7	7	7
P value	0.234	0.695	0.971	0.999	0.0006	0.004
**V261L**	5.50 ± 0.73^{NS}	–39.82 ± 1.68^{NS}	213.9 ± 4.4^{NS}	112.9 ± 1.2^{NS}	1.58 ± 0.03^{NS}	2.86 ± 0.13^{NS}
n	8	8	8	8	8	8
P value	0.831	0.342	0.858	0.902	0.779	0.268
**R1319Q**	6.00 ± 0.73^{NS}	–34.31 ± 1.30^{NS}	203.1 ± 11.4^{NS}	119.6 ± 1.4	1.67 ± 0.08^{NS}	3.03 ± 0.18^{NS}
n	6	6	6	6	6	6
P value	0.996	0.726	0.999	0.002**	0.241	0.114
**Q383E**	8.333 ± 0.61^{NS}	–30.93 ± 1.38*	209.4 ± 6.7^{NS}	123.5 ± 1.8****	1.61 ± 0.09^{NS}	3.41 ± 0.39**
n	6	6	6	6	6	6
P value	0.179	0.040	0.992	<0.0001	0.618	0.004
**E1321K**	6.66 ± 0.66^{NS}	–37.03 ± 1.31^{NS}	209.7 ± 8.5^{NS}	111.2 ± 1.9 ^{NS}	1.92 ± 0.21*	3.83 ± 0.63*
n	6	6	6	6	6	6
P value	0.925	0.968	0.729	0.999	0.027	0.011
**Q1531K**	6.33 ± 0.80^{NS}	–34.96 ± 1.98^{NS}	193.1 ± 4.5^{NS}	121.4 ± 2.5**	1.51 ± 0.08^{NS}	3.08 ± 0.23^{NS}
n	6	6	6	6	6	6
P value	0.999	0.643	0.292	0.003	0.989	0.258
**E1211K**	5.20 ± 0.48^{NS}	–45.03 ± 0.21***	164.4 ± 15.3*	100.9 ± 2.9**	2.51 ± 0.07****	5.81 ± 0.47****
n	5	5	5	5	5	5
P value	0.361	0.0001	0.011	0.0028	<0.0001	<0.0001
**D195G**	10.33 ± 0.61***	–32.93 ± 1.26*	190.6 ± 9.6^{NS}	112.8 ± 1.4^{NS}	1.61 ± 0.04^{NS}	2.88 ± 0.12^{NS}
n	6	6	6	6	6	6
P value	0.0003	0.048	0.437	0.799	0.151	0.201

The first action potential elicited by a current step 2 pA above rheobase was analysed. Firing was elicited by depolarizing step current stimuli in 2-pA increments. In the axon initial segment model, the virtual Nav1.6 channel conductance (gNav1.6) and the virtual potassium channel conductance (gKv) values were set to $gNa_V1.6 = 0$ and $gK_V = 2$. Statistically significant differences between the action potential characteristics of the wild-type (WT) and mutant channels were determined using one-way ANOVA followed by Dunnett's post-hoc test ($^{*}P < 0.05$, $^{**}P < 0.01$, $^{***}P < 0.001$, and $^{****}P < 0.0001$). Data are mean ±SEM. Abbreviations: n, number of independent experiments; NS, statistically not significant difference compared to WT.

Supplementary Table 7. Action potential firing activity during synaptic current stimulation in dynamic action potential clamp experiments implementing $Na_v1.2$ variant current and virtual conductance settings of $gNa_v1.6 = 0/gK_v = 1$.

Variant	Effects of scaled excitatory to inhibitory conductance ratios (g_e/g_i) on firing frequency (Hz)				
	$g_e/g_i = 1$	$g_e/g_i = 1.5$	$g_e/g_i = 2$	$g_e/g_i = 2.5$	$g_e/g_i = 3$
**WT**	0.12 ± 0.08	0.25 ± 0.25	0.84 ± 0.27	1.47 ± 0.29	1.55 ± 0.22
n	10	10	10	10	10
P value	–	–	–	–	–
**R1629L**	2.04 ± 0.87**	4.52 ± 1.22****	5.25 ± 1.07****	2.92 ± 0.8^{NS}	0.50 ± 0.20^{NS}
n	5	5	5	5	5
P value	0.007	<0.0001	<0.0001	0.070	0.283
**#E999K**	NF^{NS}	0.42 ± 0.22^{NS}	0.93 ± 0.23^{NS}	2.77 ± 0.63^{NS}	6.8 ± 0.62****
n	4	4	4	4	4
P value	0.999	0.997	0.999	0.176	<0.0001
**R856Q**	0.43 ± 0.21^{NS}	1.66 ± 0.60^{NS}	3.16 ± 0.6**	3.57 ± 0.66**	1.7 ± 0.4^{NS}
n	4	4	4	4	4
P value	0.979	0.119	0.002	0.007	0.998
**K905N**	NF^{NS}	0.12 ± 0.06^{NS}	0.52± 0.22^{NS}	2.4 ± 0.60^{NS}	1.45 ± 0.38^{NS}
n	5	50.6	5	5	5
P value	0.999	0.999	0.969	0.401	0.999
**#A263V**	NF^{NS}	0.33 ± 0.12^{NS}	3.44 ± 1.04*	5.01 ± 1.19***	9.19 ± 0.87****
n	8	8	8	8	8
P value	0.999	0.999	0.01	0.0002	<0.0001
**#V1325I**	0.60 ± 0.32^{NS}	3.33 ± 1.13**	5.03 ± 1.47***	7.48± 1.83****	6.6 ± 1.60**
n	5	5	5	5	5
P value	0.998	0.009	0.0002	<0.0001	0.0035
**V261L**	0.37 ± 0.22^{NS}	0.72 ± 0.31^{NS}	1.71 ± 0.25^{NS}	2.94 ± 0.34^{NS}	2.36 ± 1.27^{NS}
n	6	6	6	6	6
P value	0.998	0.987	0.846	0.404	0.878
**#R1319Q**	0.13 ± 0.09^{NS}	0.10 ± 0.06^{NS}	1.71 ± 0.75^{NS}	2.55 ± 0.66^{NS}	3.84 ± 0.93*
n	7	7	7	7	7
P value	>0.999	0.999	0.819	0.658	0.047
**#Q383E**	NF^{NS}	NF^{NS}	NF^{NS}	0.67 ± 0.59^{NS}	1.59 ± 1.0^{NS}
n	6	6	6	6	6
P value	0.999	0.998	0.861	0.883	>0.999
**E1321K**	NF^{NS}	0.06 ± 0.06^{NS}	0.60 ± 0.31^{NS}	2.61 ± 0.39 ^{NS}	3.08 ± 0.66*
n	5	5	5	5	5
P value	0.970	0.931	0.888	0.088	0.015
**#Q1531K**	NF^{NS}	0.11 ± 0.11^{NS}	0.25 ± 0.21^{NS}	1.41 ± 0.32 ^{NS}	3.46 ± 0.44***
n	7	7	7	7	7
P value	0.963	0.950	0.431	0.987	0.0006
**#E1211K**	NF^{NS}	NF^{NS}	0.05 ± 0.05^{NS}	0.10 ± 0.07**	0.35 ± 0.25*
n	4	4	4	4	4
P value	0.956	0.825	0.168	0.007	0.021
**#D195G**	NF^{NS}	NF^{NS}	NF^{NS}	0.05 ± 0.05**	0.05 ± 0.05***
n	6	6	6	6	6
P value	0.943	>0.778	0.076	0.001	0.0006

Statistically significant differences between the wild-type (WT) and mutant channels were determined using two-way ANOVA, followed by Dunnett's post-hoc test ($^{*}P < 0.05$, $^{**}P < 0.01$, $^{***}P < 0.001$, and $^{****}P < 0.0001$). $^{\#}P < 0.05$ at $g_e/g_i \geq 4$ (see Fig. 4b). Data are mean ±SEM. Abbreviations: n, number of independent experiments; NF, no firing; NS, statistically not significant difference compared to WT.

Supplementary Table 8. Biophysical characteristics of Na$_V$1.2 variants in voltage clamp (VC) experiments; the predicted neuronal excitability in dynamic action potential clamp (DAPC) experiments, and Na$_V$1.2 variant gain-of-function (GoF) or loss-of-function (LoF) effects predicted by the Web-based in silico model.

**Phenotypic group**	**Variant**	**VC analysis**<br>Selected biophysical characteristics relative to wild-type				**DAPC analysis**	**Function prediction with a Web-based model§**
		Voltage dependence of activation, *shifted?*	Voltage dependence of inactivation, *shifted?*	Recovery from fast inactivation, *changed?*	Persistent current at –30 mV, *present?*	Predicted neuronal excitability relative to wild-type	GoF or LoF effect (probability)
**Early-infantile-severe**	R1629L	yes, hyperpolarizing	yes, hyperpolarizing	no	no	GoF	GoF♣ (0.55)
	E999K	yes, hyperpolarizing	yes, hyperpolarizing	yes, slower	yes	GoF	LoF♣ (0.69)
	R856Q	yes, hyperpolarizing	yes, hyperpolarizing	no	yes	GoF	LoF♣ (0.57)
	K905N	no	yes, hyperpolarizing	yes, slower	no	no change	LoF♣ (0.6)
	[#]K905N+β_2	yes, hyperpolarizing	yes, depolarizing	no	NT	GoF	N/A
	R1882Q*	yes, hyperpolarizing	yes, depolarizing	no	yes	GoF	GoF♣ (0.81)
**Early-infantile-variable**	A263V	yes, hyperpolarizing	yes, depolarizing	yes, faster	yes	GoF	GoF♣ (0.73)
	V1325I	yes, hyperpolarizing	yes, depolarizing	no	yes	GoF	GoF (0.9)
	V261L	yes, hyperpolarizing	yes, hyperpolarizing	yes, faster	no$^{\Delta}$	GoF	GoF (0.82)
	R1319Q	no	no	no	no$^{\Delta}$	GoF	GoF♣ (0.56)
	Q383E	yes, depolarizing	yes, depolarizing	yes, faster	no$^{\Delta}$	GoF	LoF (0.73)
**Early-infantile-benign**	E1321K	no	yes, depolarizing	yes, faster	no$^{\Delta}$	GoF	GoF♣ (0.82)
	Q1531K	no	yes, depolarizing	yes, faster	yes	GoF	LoF♣ (0.71)
	L1563V*	yes, depolarizing	yes, depolarizing	yes, faster	no	GoF	LoF♣ (0.76)
**Later-onset**	D195G	yes, depolarizing	yes, hyperpolarizing	yes, slower	no	LoF	LoF (0.73)
	E1211K	yes, hyperpolarizing	yes, hyperpolarizing	yes, slower	no	LoF	LoF♣ (0.8)
	R853Q*	yes, depolarizing	yes, hyperpolarizing	no	no	LoF	LoF♣ (0.79)

Abbreviations: VC, voltage clamp; DAPC, dynamic action potential clamp; GoF, gain-of-function; LoF, loss-of-function; *, missense variants characterized in our previous study[13]; $^{\Delta}$, persistent current is present at membrane voltages more positive than –30 mV; §, machine learning–based statistical model, trained on selected protein features of published LoF and GoF variants[14]; ♣, unreliable functional prediction, because variant is part of the training data[14]; N/A, not applicable; NT, not tested; [#]Biophysical characteristics and DAPC prediction relative to control variant (wild-type α1 subunit) co-expressed with β_2 subunit.

Supplementary Table 9. The effect of β_2 subunit co-expression on the biophysical characteristics of the wild-type and K905N Na_V1.2 channel variants in voltage clamp experiments.

Variant	Biophysical property			
	**I_{Na} density at −10 mV (pA/pF)**	**$V_{0.5,act}$ (mV)**	**$V_{0.5,inact}$ (mV)**	**τ recovery (ms)**
**WT***	426.1 ± 72^{NS}	−17.32 ± 0.57^{**}	−49.76 ± 0.79^{**}	1.11 ± 0.02^{*}
n	10	10	10	9
P value	0.839	0.001	0.001	0.031
**WT + β2**	487.8 ± 73	−13.94 ± 0.64	−53.52 ± 0.62	1.38 ± 0.10
n	14	12	14	12
P value	-	-	-	-
**K905 + β2**	498.4 ± 92^{NS}	−20.21 ± 0.56^{****}	−50.41 ± 0.65^{**}	1.31 ± 0.05^{NS}
n	15	14	15	14
P value	0.993	<0.0001	0.003	0.0001

WT*, CHO cells transfected with wild-type α_1 subunit alone; WT + β_2, CHO cells transfected with wild-type α_1 and β_2 subunits; K905N + β_2, CHO cells transfected with K905N α_1 and β_2 subunits. Statistically significant differences between WT + β_2 and K905N + β_2 or WT* channels were determined using one-way ANOVA, followed by Dunnett's post-hoc test ($^{*}P < 0.05$, $^{**}P < 0.01$, and $^{****}P < 0.0001$); NS, statistically not significant difference compared to WT + β_2. Data are represented as mean ±SEM. Abbreviations: n, number of independent experiments; I_{Na}, sodium current; $V_{0.5,act}$, membrane potential for half-maximal activation; $V_{0.5,inact}$, membrane potential for half-maximal inactivation; τ recovery, time constant of recovery from fast inactivation.

Supplementary Table 10. Action potential firing activity in dynamic action potential clamp experiments implementing wild-type or K905N Na_V1.2 channel variants co-expressed with β_2 subunit (WT + β_2 and K905N + β2, respectively), or wild-type subunit alone (WT*).

Variant	Effects of step current (I_{st}) magnitude on firing frequency (Hz)					
	**I_{st} = 4 nA**	**I_{st} = 8 nA**	**I_{st} = 10 nA**	**I_{st} = 12 nA**	**I_{st} = 14 nA**	**I_{st} = 16 nA**
**WT***	0.12 ± 0.12^{NS}	3.25 ± 0.78^{NS}	7.00 ± 1.32^{****}	6.62 ± 1.24^{***}	5.14 ± 1.08^{*}	4.28 ± 1.0^{NS}
n	7	7	7	7	7	7
P value	0.998	0.182	<0.0001	0.0005	0.023	–
**WT + β2**	0.08 ± 0.08	1.75 ± 0.32	2.75 ± 0.44	3.25 ± 0.42	2.83 ± 0.34	2.45 ± 0.28
n	10	10	10	10	10	10
P value	-	-	-	-	-	-
**K905 + β2**	0.18 ± 0.12^{NS}	4.27 ± 1.23^{**}	9.18 ± 1.60^{****}	9.81 ± 1.97^{****}	6.27 ± 1.13^{***}	4.81 ± 0.82^{*}
n	8	8	8	8	8	8
P value	0.991	0.008	<0.0001	<0.0001	0.0002	0.014

Dynamic action potential clamp experiments were performed with virtual conductance settings of $gNa_V1.6 = 0/gK_V = 2$. The input-output relationships of these experiments are shown in Figure 5f (main manuscript). Differences in firing rates at each step current stimulus were determined relative to WT + β_2 using multiple comparisons in two-way ANOVA, followed by Dunnett's post-hoc test ($^{*}P < 0.05$, $^{**}P < 0.01$, and, $^{****}P < 0.0001$); NS, statistically not significant difference compared to WT + β_2. Data are represented as mean ±SEM; n, number of experiments.

Supplementary Table 11. The effect of β_2 subunit co-expression on action potential characteristics in dynamic action potential clamp experiments implementing wild-type or K905N $Na_v1.2$ channel variants.

Variant	Rheobase (pA)	Threshold (pA)	Upstroke velocity (dV/dt)	Amplitude (mV)	Width (ms)	Decay time (ms)
**WT***	6.67 ± 0.42^{NS}	–36.26± 0.63^{NS}	199.1 ± 8.2^{NS}	113.5 ± 2.27^{NS}	1.46 ± 0.07^{NS}	2.12 ± 0.19^{NS}
**n**	7	7	7	7	7	7
**P value**	0.512	0.265	0.958	0.530	0.562	0.997
**WT + β_2**	7.40 ± 0.52	–34.69± 0.49	197.0 ± 5.2	115.6 ± 1.33	1.40 ± 0.04	2.11 ± 0.08
**n**	10	10	10	10	10	10
**P value**	-	-	-	-	-	-
**K905N + β_2**	6.60 ± 0.52^{NS}	–37.73± 1.07*	197.6 ± 4.9^{NS}	118.0 ± 0.8^{NS}	1.37 ± 0.02^{NS}	2.07 ± 0.05^{NS}
**n**	8	8	8	8	8	8
**P value**	0.427	0.013	0.996	0.418	0.849	0.952

Dynamic action potential clamp experiments were performed with virtual conductance settings of $gNa_v1.6 = 0/gK_v = 2$. Statistically significant differences between the WT* or K905N + β_2 channels and WT + β_2 were determined using one-way ANOVA, followed by Dunnett's post-hoc test ($^{*}P < 0.05$); NS, statistically not significant difference compared to WT + β_2. Data are represented as mean ± SEM. Abbreviations: n, number of independent experiments; WT*, CHO cells transfected with wild-type α_1 subunit alone; WT + β_2, CHO cells transfected with wild-type α_1 and β_2 subunits; K905N + β_2, CHO cells transfected with K905N α_1 and β_2 subunits.

Supplementary Table 12. Site-directed mutagenesis primers

**Primers**	**Sequences (5' to 3' direction)**
R1629L-For	CTGTTCCGAGTGATCCTTCTTGCCAGGATTGGC
R1629L-Rev	GCCAATCCTGGCAAGAAGGATCACTCGGAACAG
E999K-For	GCTGCCACTGATGATGATAACAAAATGAATAATCTCCAGATTG
E999K-Rev	CAATCTGGAGATTATTCATTTTGTTATCATCATCAGTGGCAGC
R856Q-For	ATCATTCCGGCTGCTCCAAGTTTTCAAGTTGGCAA
R856Q-Rev	TTGCCAACTTGAAAACTTGGAGCAGCCGGAATGAT
K905N-For	CGGCATGCAGCTCTTTGGTAACAGCTACAAAGAATGTGTCTG
K905N-Rev	GCAGACACATTCTTTGTAGCTGTTACCAAAGAGCTGCATGCCG
A263V-For	GTTCTGTCTAAGCGTGTTTGTGCTAATAGGATTGCAGTT
A263V-Rev	AACTGCAATCCTATTAGCACAAACACGCTTAGACAGAAC
V1325I-For	CCCGGTTTGAAGGAATGAGGATTGTTGTAAATGCTCTTTTA
V1325I-Rev	TAAAAGAGCATTTACAACAATCCTCATTCCTTCAAACCGGG
V261L-For	CTGTGTTCTGTCTAAGCCTGTTTGCGCTAATAGGA
V261L-Rev	TCCTATTAGCGCAAACAGGCTTAGACAGAACACAG
R1319Q-For	ACTGAGAGCTTTGTCCCAGTTTGAAGGAATGAGGG
R1319Q-Rev	CCCTCATTCCTTCAAACTGGGACAAAGCTCTCAGT
Q383E-For	CTTATTTCGTCTCATGACTGAAGACTTCTGGGAAAACCT
Q383E-Rev	AGGTTTTCCCAGAAGTCTTCAGTCATGAGACGAAATAAG
E1321K-For	AGAGCTTTGTCCCGGTTTAAAGGAATGAGGGTTGT
E1321K-Rev	ACAACCCTCATTCCTTTAAACCGGGACAAAGCTCT
Q1531K-For	ATGGTCTTTGATTTTGTAACCAAAAAAGTCTTTGATATCAGCATCATG
Q1531K-Rev	CATGATGCTGATATCAAAGACTTTTTTGGTTACAAAATCAAAGACCAT
E1211K-For	TAGTGGAGCACAATTGGTTCAAAACCTTCATTGTCTTCATG
E1211K-Rev	CATGAAGACAATGAAGGTTTTGAACCAATTGTGCTCCACTA
D195G-For	GGATCCATGGAATTGGTTGGGTTTCACAGTCATTACTTTTG
D195G-Rev	CAAAAGTAATGACTGTGAAACCCAACCAATTCCATGGATCC

Abbreviations: For, forward; Rev, reverse (Rev)

SUPPLEMENTARY REFERENCES

1 Pan, X. *et al.* Molecular basis for pore blockade of human Na^+ channel $Na_v1.2$ by the μ-conotoxin KIIIA. *Science* **363**, 1309-1313, doi:10.1126/science.aaw2999 (2019).

2 Clairfeuille, T. *et al.* Structural basis of α-scorpion toxin action on Na_v channels. *Science* **363**, doi:10.1126/science.aav8573 (2019).

3 Ahern, C. A., Payandeh, J., Bosmans, F. & Chanda, B. The hitchhiker's guide to the voltage-gated sodium channel galaxy. *J Gen Physiol* **147**, 1-24, doi:10.1085/jgp.201511492 (2016).

4 Syrbe, S. *et al.* Phenotypic Variability from benign infantile epilepsy to Ohtahara syndrome associated with a novel mutation in *SCN2A*. *Mol Syndromol* **7**, 182-188, doi:10.1159/000447526 (2016).

5 Yarov-Yarovoy, V. *et al.* Structural basis for gating charge movement in the voltage sensor of a sodium channel. *Proc Natl Acad Sci U S A* **109**, E93-102, doi:10.1073/pnas.1118434109 (2012).

6 Gallivan, J. P. & Dougherty, D. A. Cation-π interactions in structural biology. *Proc Natl Acad Sci U S A* **96**, 9459-9464, doi:10.1073/pnas.96.17.9459 (1999).

7 Garrido, J. J. *et al.* Dynamic compartmentalization of the voltage-gated sodium channels in axons. *Biol Cell* **95**, 437-445, doi:10.1016/s0248-4900(03)00091-1 (2003).

8 Chahine, M., Ziane, R., Vijayaragavan, K. & Okamura, Y. Regulation of Na_v channels in sensory neurons. *Trends Pharmacol Sci* **26**, 496-502, doi:10.1016/j.tips.2005.08.002 (2005).

9 Camacho, J. A. *et al.* Modulation of $Na_v1.5$ channel function by an alternatively spliced sequence in the DII/DIII linker region. *J Biol Chem* **281**, 9498-9506, doi:10.1074/jbc.M509716200 (2006).

10 Muroi, Y., Arcisio-Miranda, M., Chowdhury, S. & Chanda, B. Molecular determinants of coupling between the domain III voltage sensor and pore of a sodium channel. *Nat Struct Mol Biol* **17**, 230-237, doi:10.1038/nsmb.1749 (2010).

11 Arcisio-Miranda, M., Muroi, Y., Chowdhury, S. & Chanda, B. Molecular mechanism of allosteric modification of voltage-dependent sodium channels by local anesthetics. *J Gen Physiol* **136**, 541-554, doi:10.1085/jgp.201010438 (2010).

12 Lauxmann, S. *et al.* Relationship of electrophysiological dysfunction and clinical severity in *SCN2A*-related epilepsies. *Hum Mutat* **39**, 1942-1956, doi:10.1002/humu.23619 (2018).

13 Berecki, G. *et al.* Dynamic action potential clamp predicts functional separation in mild familial and severe de novo forms of *SCN2A* epilepsy. *Proc Natl Acad Sci U S A* **115**, E5516-E5525, doi:10.1073/pnas.1800077115 (2018).

14 Heyne, H. O. *et al.* Predicting functional effects of missense variants in voltage-gated sodium and calcium channels. *Sci Transl Med* **12**, doi:10.1126/scitranslmed.aay6848 (2020).
