# Supplementary material for: Functional correlates of clinical phenotype and severity in recurrent SCN2A variants
Source: Commun Biol. 2022 May 30;5:515. doi: 10.1038/s42003-022-03454-1 (PMC9151917; doi:10.1038/s42003-022-03454-1)
Supplement: Supplementary file 3 — Description of Additional Supplementary Files [file 42003_2022_3454_MOESM3_ESM.pdf]

Description of Additional Supplementary Files

File name: Supplementary Data 1
Description: Clinical features of all individuals included in the study.

File name: Supplementary Data 2
Description: The biophysical characteristics and the predicted functional impact of all recurrent variants included in this study.

File name: Supplementary Data 3
Description: The source data points for Figures 2b-f, 3b-e, 4b-e, 5c-d, and 5f.
