# Supplementary material for: Functional correlates of clinical phenotype and severity in recurrent SCN2A variants
Source: Commun Biol. 2022 May 30;5:515. doi: 10.1038/s42003-022-03454-1 (PMC9151917; doi:10.1038/s42003-022-03454-1)
Supplement: Supplementary file 7 — Reporting Summary [file 42003_2022_3454_MOESM7_ESM.pdf]

nature research

Corresponding author(s): Geza Berecki

Last updated by author(s): Apr 21, 2022

Reporting Summary

Nature Research wishes to improve the reproducibility of the work that we publish. This form provides structure for consistency and transparency in reporting. For further information on Nature Research policies, see our Editorial Policies and the Editorial Policy Checklist.

Statistics

For all statistical analyses, confirm that the following items are present in the figure legend, table legend, main text, or Methods section.

n/a	Confirmed	
☐	☒	The exact sample size (n) for each experimental group/condition, given as a discrete number and unit of measurement
☐	☒	A statement on whether measurements were taken from distinct samples or whether the same sample was measured repeatedly
☐	☒	The statistical test(s) used AND whether they are one- or two-sided<br>*Only common tests should be described solely by name; describe more complex techniques in the Methods section.*
☒	☐	A description of all covariates tested
☐	☒	A description of any assumptions or corrections, such as tests of normality and adjustment for multiple comparisons
☐	☒	A full description of the statistical parameters including central tendency (e.g. means) or other basic estimates (e.g. regression coefficient) AND variation (e.g. standard deviation) or associated estimates of uncertainty (e.g. confidence intervals)
☐	☒	For null hypothesis testing, the test statistic (e.g. F, t, r) with confidence intervals, effect sizes, degrees of freedom and P value noted<br>*Give P values as exact values whenever suitable.*
☒	☐	For Bayesian analysis, information on the choice of priors and Markov chain Monte Carlo settings
☒	☐	For hierarchical and complex designs, identification of the appropriate level for tests and full reporting of outcomes
☒	☐	Estimates of effect sizes (e.g. Cohen's d, Pearson's r), indicating how they were calculated

Our web collection on statistics for biologists contains articles on many of the points above.

Software and code

Policy information about availability of computer code

Data collection	not applicable
Data analysis	Electrophysiology data were analysed using Clampfit 9.2 (Molecular Devices). Statistical evaluation of data was performed using GraphPad Prism version 9 (La Jolla, CA, USA). Figures were created using Origin 9.0 (Microcal Software Inc., Northampton, MA).

For manuscripts utilizing custom algorithms or software that are central to the research but not yet described in published literature, software must be made available to editors and reviewers. We strongly encourage code deposition in a community repository (e.g. GitHub). See the Nature Research guidelines for submitting code & software for further information.

Data

Policy information about availability of data

All manuscripts must include a data availability statement. This statement should provide the following information, where applicable:

- Accession codes, unique identifiers, or web links for publicly available datasets
- A list of figures that have associated raw data
- A description of any restrictions on data availability

The datasets generated during and/or analysed during the current study are available from the corresponding author on reasonable request of any qualified investigator for purposes of replicating procedures and results.

Field-specific reporting

Please select the one below that is the best fit for your research. If you are not sure, read the appropriate sections before making your selection.

☒ Life sciences ☐ Behavioural & social sciences ☐ Ecological, evolutionary & environmental sciences

For a reference copy of the document with all sections, see nature.com/documents/nr-reporting-summary-flat.pdf

Life sciences study design

All studies must disclose on these points even when the disclosure is negative.

Sample size	For the analysis of the biophysical properties of heterologously expressed Nav1.2 channel variants, we collected data from at least three independent transfections. For each variants, a minimum of six cells were assessed.
Data exclusions	Chinese hamster ovarian (CHO) cells of 12–25 pF cell capacitance and expressing 2–10 nA peak Nav1.2 currents (INa) were included in the study; cells expressing small INa (< 2 nA, ~20 % of cells) or large INa (>10 nA, ~20 %) were excluded, according to a previously published protocol (Berecki et al. 2018. Proc Natl Acad Sci U S A 115, E5516-E5525, doi:10.1073/pnas.1800077115).
Replication	The biophysical data included in this study is reproducible. This has been confirmed in transfections repeated at least three times for each individual variant included in the study. Notably, the biophysical data obtained for the wild-type (control) Nav1.2 channel in this study is similar to our previously published data (Berecki et al. 2018. Proc Natl Acad Sci U S A 115, E5516-E5525, doi:10.1073/pnas.1800077115).
Randomization	Individual channel variants (mutants) were simultaneously studied with the wild-type Nav1.2 channel to maintain identical experimental conditions.
Blinding	Blinding was not used in this study.

Reporting for specific materials, systems and methods

We require information from authors about some types of materials, experimental systems and methods used in many studies. Here, indicate whether each material, system or method listed is relevant to your study. If you are not sure if a list item applies to your research, read the appropriate section before selecting a response.

Materials & experimental systems

n/a	Involved in the study	
☒	☐	Antibodies
☐	☒	Eukaryotic cell lines
☒	☐	Palaeontology and archaeology
☒	☐	Animals and other organisms
☐	☒	Human research participants
☐	☒	Clinical data
☒	☐	Dual use research of concern

Methods

n/a	Involved in the study	
☒	☐	ChIP-seq
☒	☐	Flow cytometry
☒	☐	MRI-based neuroimaging

Eukaryotic cell lines

Policy information about cell lines

Cell line source(s)	Chinese hamster ovarian (CHO) cells.
Authentication	CHO cells are commercially available for research and our cells were not specifically authenticated.
Mycoplasma contamination	The CHO cells used tested negative for mycoplasma contamination. In our laboratory, mycoplasma testing is carried out every three months.
Commonly misidentified lines (See ICLAC register)	not applicable

Human research participants

Policy information about studies involving human research participants

Population characteristics	This information is provided detailed in the Supplementary Data 1 file.
Recruitment	The medical literature, the SCN2A International Natural History Study (NHS) database, the Florey Institute's Ion Channels

Recruitment	Laboratory database, and the Simons Searchlight database (SSDb) (https://www.sfari.org/resource/simons-searchlight/) were searched to identify all recurrent SCN2A variants with clinical information available on affected individuals. Details on patient selection are provided in the main manuscript and Supplementary Methods.
Ethics oversight	The study was approved by the Human Research Ethics Committees of the Royal Children's Hospital and Austin Health Melbourne, University Medical Center Groningen, State Medical Association of Berlin, and the Simons Foundation. Written informed consent was obtained for all individuals whose previously unpublished clinical data is presented here.

Note that full information on the approval of the study protocol must also be provided in the manuscript.

Clinical data

Policy information about clinical studies

All manuscripts should comply with the ICMJE guidelines for publication of clinical research and a completed CONSORT checklist must be included with all submissions.

Clinical trial registration	Not applicable
Study protocol	Clinical information was collected as described in Methods of the manuscript.
Data collection	See above (recruitment)
Outcomes	Not applicable
